# Case Report: Cytoreductive surgery as a therapeutic attempt in the treatment of extramedullary plasmacytoma

**DOI:** 10.3389/fsurg.2026.1830271

**Published:** 2026-05-20

**Authors:** Jakub Halámek, Ivan Špička, Zdeněk Krška, Kristian Chrz

**Affiliations:** 1 1st Surgical Department, General University Hospital, Prague, Czechia; 2 1st Medical Faculty, Charles University, Prague, Czechia; 3 1st Department of Medicine and Hematology, General University Hospital, Prague, Czechia

**Keywords:** case report, cytoreductive surgery, extramedullary plasmacytoma (EP), multiple myeloma, wound healing

## Abstract

**Introduction:**

Multiple myeloma is a haematological malignancy based on pathological monoclonal proliferation of plasma cells, which usually occurs in the bone marrow. Known aspects of its symptomatology include anaemia, renal failure, hypercalcaemia, osteolytic bone lesions and potentially consequences of AL amyloidosis. Diagnostics is multimodal and relies on laboratory and imaging methods of conventional radiology and nuclear medicine. Treatment is then the domain of the haematologist-oncologist. One form of this disease is extramedullary plasmacytoma, i.e., a solitary or multiple focus of multiple myeloma, which is not anatomically associated with bone marrow. This case report focuses on a patient with an advanced form of extramedullary plasmacytoma, who underwent partial cytoreductive surgery as a therapeutic attempt after exhausting all lines of conventional treatment.

**Case presentation:**

A 67-year-old male patient with an advanced form of extramedullary plasmacytoma was referred by his attending haematologist to the 1st Department of Surgery of General University Hospital in Prague of his attending haematologist to assess the possibility of extirpation of EMP lesions, due to the exhaustion of systemic therapy options. The aim of considered intervention was one of the macroscopically manifested soft tissue lesions of extramedullary plasmacytoma, which appeared suitable for surgical removal. After comprehensive preoperative assessment the elective surgical cytoreduction of the large lesion of extramedullary plasmacytoma above the right scapula was successfully performed. From the surgeon's perspective, the postoperative course was quite favourable without periprocedural complications, which ultimately led to the complete healing of the surgical wound. Despite achieving the primary surgical goal and the ongoing systemic treatment, the underlying disease developed to the stage beyond all possible therapeutic options, which ultimately led to the death of the patient.

**Discussion:**

In conclusion, despite the overall result of this case, it can be said about this therapeutic attempt that, assuming careful consideration of indications within the framework of interdisciplinary cooperation, considering all eventualities, this may be a relatively undemanding and safe procedure that does not exceed the capabilities of a standard surgical facility, is reproducible and potentially available as an addition to conventional treatment of extramedullary plasmacytoma in highly specific cases.

## Introduction

Multiple myeloma (MM) is a haematological malignancy based on pathological monoclonal proliferation of plasma cells, which usually occurs in the bone marrow. One of the diagnostic criteria is the presence of more than 10% of these cells in a bone marrow biopsy sample (1).

With an incidence of 6/100,000 (over 35,750 new cases worldwide in 2024), MM is the second most common haematological malignancy and represents 13% of them all (2, 3). The median age of patients at the time of diagnosis ranges from 65 to 70 years (3, 4). With early detection and the use of current comprehensive therapeutic procedures, the median survival rate is around 8 years (3, 5).

One form of this disease, especially in advanced stages, is extramedullary plasmacytoma (EMP), i.e., a solitary or multiple focus of multiple myeloma, which is not anatomically associated with the bone marrow (1–3). Of the reported incidence of MM, 0.5%–5.2% accounts for primary detection with the presence of some form of EMP, however, in the event of relapse, extramedullary involvement is present in 5%–30% of cases (5). These lesions mostly occur in the soft tissues and structures of head and neck, more specifically in the upper parts of respiratory and digestive system, but also can be located in the liver, pancreas, kidneys, lower parts of respiratory and digestive system, heart, central nervous system or extremities (5, 6).

The clinical manifestation is quite rich and can be highly variable over the course of the disease. A tetrad of symptoms and paraclinical manifestations characterised by the acronym CRAB—calcium (hypercalcaemia), renal insufficiency (result of light chains of paraprotein precipitation tubulopathy), normocytic normochromic anaemia, osteolytic bone lesions with significant risk of pathological fractures is well known (4, 7). Other consequences of the underlying disease could be also manifestation of AL amyloidosis, physiological haematopoietic restriction, and consequences of the local presence and growth of EMP lesions (3, 4, 7).

In terms of extent, dissemination, and anatomical conditions of the lesions six units are defined: solitary plasmacytoma without bone marrow infiltration, solitary plasmacytoma with minimal bone marrow infiltration (<10% plasma cells), bone-associated plasmacytoma, bone-unrelated plasmacytoma, organ-infiltrating plasmacytoma, and plasma cell leukaemia (1, 3).

Imaging methods (skeletal radiography, low-dose CT, MRI, PET/CT) and laboratory diagnostic methods (electrophoresis of plasma proteins, blood count, biochemistry, cytological, cytogenetics methods etc.) are used for further examination to confirm the diagnosis, monitor the course of the disease, including response to therapy, and detect potential complications at an early stage (1, 7). Some of these methods also have significant prognostic value.

Therapy and follow-up are primarily the domain of the haematologist-oncologist. The main treatment modality is biological therapy, followed by chemotherapy, autologous stem cell transplantation, radiotherapy, and multimodal supportive care (1–8). The role of the surgeon in this case is rather complementary in the sense of comprehensive care for pathological fractures, however, several case reports and systemic reviews describing the surgical removal of EMP lesions as a part of the curative treatment were published (6, 7, 9–11).

The subject of this case report was a patient with advanced extramedullary plasmacytoma within multiple myeloma, in whom, due to the unfavourable course of the disease and the exhaustion of conventional therapeutic options, partial cytoreductive surgery (CRS) was considered and performed as the last and quite unusual therapeutic attempt. The aim of this paper is to present a modest experience with the care of a patient with extramedullary plasmacytoma in an indication that is still at least significantly rare for a surgeon, and to ask whether this procedure improved the prognosis or quality of life of the patient.

## Case presentation

### First contact

A 67-year-old male patient presented to the 1st Department of Surgery on 4 June 2025 on the recommendation of his attending haematologist to assess the possibility of surgical extirpation of EMP lesions due to exhaustion of systemic therapy options. The timeline of the main diagnostic and therapeutic events during surgical care is summarized in Table 1. Specifically, at that time, he was undergoing 7th-line systemic treatment with the bispecific antibody talquetamab, which targets the GPRC5D receptor and CD3 receptor on T lymphocytes. Its effect was not significant, but not zero either—there was a limitation of extramedullary lesions and stabilisation of their size, alleviation of pain associated with rapid growth of the lesions, and disappearance of B symptoms. However, from the few clinical analyses and brief clinical practice, it is known that the efficacy of bispecific antibodies and other treatment modalities is limited in EMP, and therefore sufficient cytoreduction cannot be expected, which also poses a risk of further disease progression (12). The presumed explanation is limited penetration of the drug into tumour tissue. At this stage of treatment, it was also clear that, according to laboratory parameters, the treatment was effective outside the EMP area. For these reasons, the main objective of the intervention considered was, albeit theoretically, an attempt at cytoreductive surgery of the tumour masses.

**Table 1:** Timeline of the main diagnostic and therapeutic events during surgical care.

Timeline of the case	
Date	Event
4 June 2025	First contact of the patient with the surgeon, first consideration of CRS options
12 June 2025	Preoperative CT scan
23 June 2025	Admission of patient to surgical department
24 June 2025	Cytoreductive surgery procedure
26 June 2025	Dismission of patient from hospital
1 July 2025	1st outpatient check-up by the surgeon
8 July 2025	2nd outpatient check-up by the surgeon
16 July – 20 August 2025	Wound healing outpatient clinic dispensary
27 September 2025	Patient deceased

**Figure 1:**
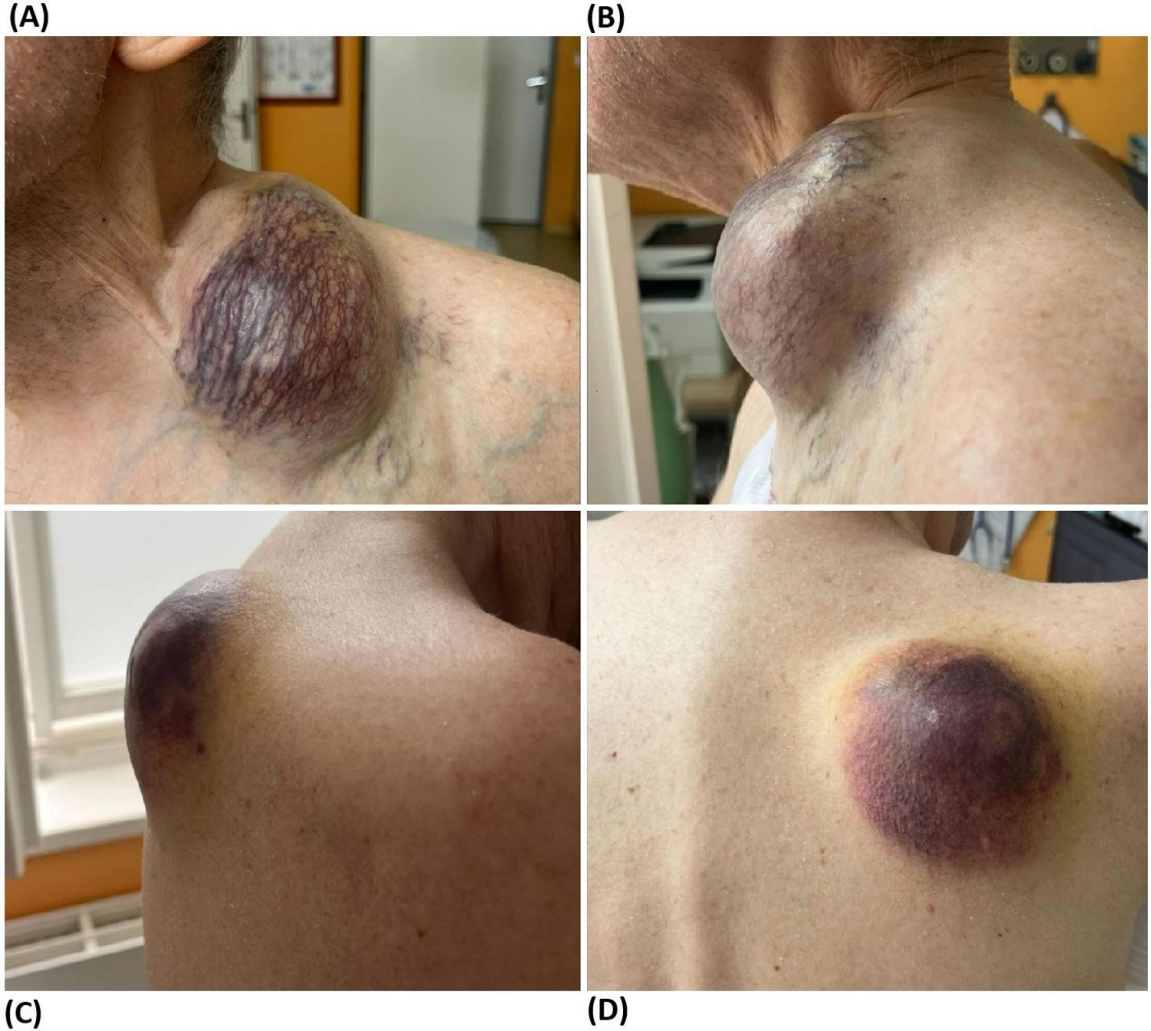
Local manifestation of EMP lesions, made 4 June 2025: **(A)** EMP lesion above the left clavicle (front view). **(B)** EMP lesion above the left clavicle (side view). **(C)** EMP lesion above the right scapula (side view). **(D)** EMP lesion above the right scapula (rear view).

The first examination by the surgeon revealed three macroscopically evident EMP lesions—the largest above the left clavicle, was relatively firmly attached to surrounding tissues, the second, above the right scapula, was quite mobile to the base, and the third was palpable in the left axilla. The skin covering the lesions was chronically indurated and permeated by a dense network of superficial telangiectasias (Figures 1A–D).

**Figure 2:**
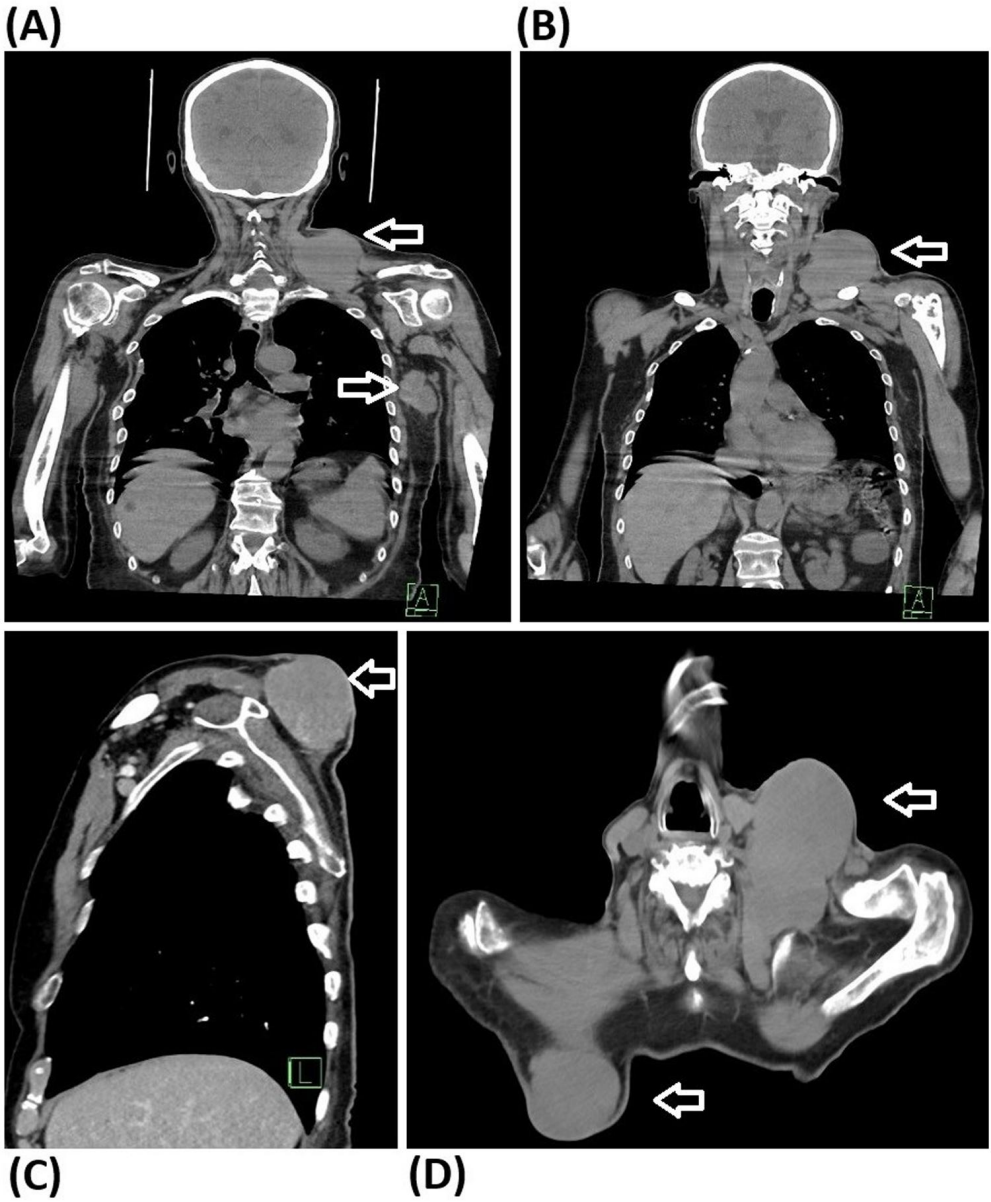
CT scan made 12 June 2025, imaging the location and extent of EMP lesions: **(A)** Coronal scan imaging the lesions above the left clavicle and in the left axilla. **(B)** Another coronal scan imaging dorsal parts of lesion above the left clavicle. **(C)** Sagittal scan imaging the well bordered lesion above the right scapula. **(D)** Axial scan imaging the lesion above the left clavicle in its extent invading to the deeper compartments of neck and chest, and the extent of lesion above the right scapula.

As part of the preoperative assessment was provided the post-contrast CT scan of the head, neck, and torso with a closer verification of the lesions. The scan taken on 12 June 2025 showed multiple soft tissue lesions, specifically above the left clavicle (98 × 71 mm), behind the right scapula (73 × 53 mm), paraesophageally on the left (17 × 10 mm) and in the left axilla (79 × 64 mm). Compared to the previous CT scan taken six months earlier, all these lesions showed an average increase approximately 20 mm (Figures 2A–D).

After certain organisational adjustments, the date of the surgical procedure was set for 24 June 2025, with admission to hospital one day earlier.

### Preoperative assessment

The following factors were considered in the comprehensive preoperative assessment. The current blood count, in particular Hb 80–90 g/L and platelets 85–100 × 10^9^/L values, allowed for surgery. On the other hand, it was necessary to consider the expected abundant vascularisation of the lesion and surrounding tissues, chronic thrombocytopenia, and antiplatelet therapy with acetylsalicylic acid, which would certainly cause greater blood loss and difficult intraoperative haemostasis. In chronic anaemia as part of the underlying disease, this could cause a serious, even life-threatening complication. To compensate potential blood loss, two transfusion units were secured, and antiplatelet therapy was discontinued seven days ahead.

Another thoroughly considered factor was the extent of the procedure. The EMP lesion above the right scapula (73 × 53 mm) appeared suitable for extirpation due to its favourable location in the subcutaneous tissue epifascially, sharp borders and relatively free mobility. The lesion above the left clavicle (98 × 71 mm) seemed not suitable for surgical removal. Its close relationship with the left internal jugular vein and left subclavian artery, local advancement and active growth on the border between the deep compartments of the neck and upper anterior mediastinum would certainly require a multidisciplinary approach involving a team of ENT specialists and thoracic surgeons. However, such a potential procedure would be exceedingly difficult to perform due to its extent, duration, high blood loss, and risk of injury of important structures, at the cost of a substantial risk of numerous periprocedural complications, even endangering the patient's life. Similar reasons, together with limited experience with the surgical approach to EMP tumour masses and the risk of iatrogenic injury to the osteolytically affected skeleton during positioning, led to the decision to perform an extirpation of the lesion in the left axilla (53 × 36 mm) at a later date. An additional argument against this time-consuming and technically demanding procedure was the patient's immunocompromised state, associated with a higher risk of infectious complications and impaired healing of the surgical wound.

The choice of surgical approach and technique for the extirpation of the selected lesion above the right scapula was also carefully considered. Given the extent of chronic skin changes above the lesion—long-term distension, multiple telangiectasias and a thin layer of subcutaneous tissue—it was clear that the procedure would require a significant loss of skin cover, with low possibility of complete primary suture. The question therefore arose as to whether, after excision, to use the technically and time-consuming creation of a sliding or rotational flap to reconstruct the resulting defect, or to perform only a partial primary suture and leave the residual defect to heal per secundam intentionem. After careful consideration, taking into account all risk factors and potential complications, it was decided to proceed with the second option. It also appeared necessary to remove the lesion as whole without fragmentation of tumour, which would lead to very difficult-to-control bleeding.

Ciprofloxacin 400 mg in 3 doses was chosen as antibiotic prophylaxis, as the patient had a history of anaphylactic reactions to penicillin antibiotics.

### Surgical procedure

The planned surgical procedure began on 24 June 2025 at 10:55 p.m. In general anaesthesia with orotracheal intubation in a stable position on his left side skin excision was performed using a modified “lazy S” incision, followed by gradual dissection of the soft tissues surrounding the tumour. Bleeding from highly vascularised tissues was successfully controlled using monopolar and bipolar coagulation. The tumour was completely separated from the fascia of the trapezius and latissimus dorsi muscles and sent for histopathological examination, which subsequently confirmed the presumed diagnosis of EMP. Subsequently, an extensive mobilisation of the edges of the resulting defect was performed, which allowed almost complete suture of the subcutaneous tissue and skin, except for a central residual defect measuring approximately 20 × 30 mm. Edges were anchored to the fascia with several sutures.

The procedure was completed on 25 June 2025 at 1:25 a.m. The patient was brought out of general anaesthesia, transported in a stable condition to the recovery room and then back to the standard ward. Blood loss in total 200-250 mL.

### Postoperative course

The early postoperative course appeared favourable on the first postoperative day. The patient had no complaints or significant pain, was cardiopulmonary compensated, eupnoeic, fully mobile, and self-sufficient. The dressing material showed no signs of sanguinolent leakage. The only expected anomaly was a decrease in haemoglobin concentration (89 → 75 g/L), however, without manifestations of anaemic syndrome. Two transfusion units were therefore applied, achieving satisfactory correction of the blood count (Hb 75 → 85 g/L).

**Figure 3:**
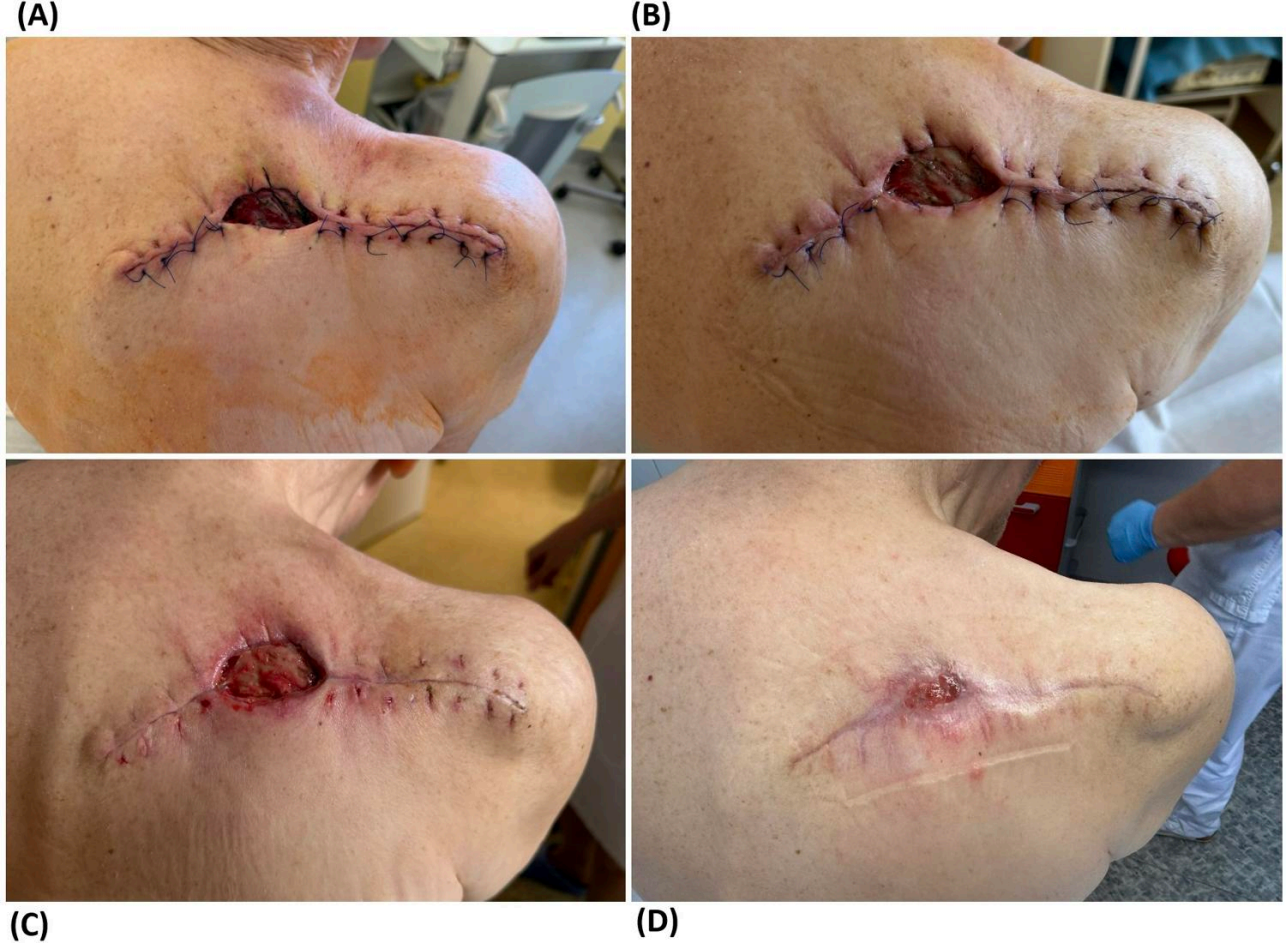
Postoperative course of surgical wound healing. **(A)** made 26 June 2025. **(B)** made 1 July 2025. **(C)** made 8 July 2025. **(D)** made 30 July 2025.

The first dressing change was performed on the second day. The dressing material was only slightly soaked with serosanguinolent secretion, and the wound itself was calm with no accentuated local signs of inflammation. The residual defect showed no signs of progression, active bleeding, or early healing disorders (Figure 3A). On the same day, the patient was discharged to home care in a compensated condition.

The first outpatient check-up by the surgeon after the procedure took place on the eighth postoperative day. The patient arrived in a compensated condition, he denied any significant difficulties or noticeable deterioration compared to his long-term condition. A central residual defect of the wound persisted, but without signs of infection or impaired healing (Figure 3B). The wound bed was covered with clean granulation tissue, and the sutures were left in place.

At this stage, moist wound healing was initiated using L-Mesitran Hydro and L-Mesitran Foam materials, the use of which proved to be greatly beneficial. At the next outpatient check-up after 7 days, the skin defect showed signs of incipient epithelialisation, with the decreasing depth due to the growth of vital granulation tissue (Figure 3C).

Subsequent wound checks were performed on a regular weekly basis at the wound healing outpatient clinic of the 1st Department of Surgery of General University Hospital. The favourable healing trend continued with progressive epithelialisation over adequately growing granulation tissue (Figure 3D), so that two months after the procedure, the defect was completely healed. On 20 August 2025, follow-up surgical care was terminated.

However, although the wound healing trend after surgical intervention was favourable, the development of the patient's underlying disease was another thing. Even with only a week between check-ups, it was clear that the plasmacytoma lesions supraclavicularly on the left and now in both axillae continued to grow expansively, with a persisting upward trend of paraprotein level (2 July 2025–10 September 2025: 12.44 → 18.16 → 30.94 → 42.74 g/L), which remained unaffected by the cytoreductive procedure, despite the ongoing systemic treatment. Given this unfavourable development of the underlying disease and the poor prognosis, further attempts at curative intervention were discontinued and the treatment was limited to palliative and supportive BSC (best supportive care). The patient died on 27 September 2025.

## Discussion

Although the therapeutic results of MM treatment are among the greatest advances in haematological oncology in the last 20 years, the disease remains incurable in a significant proportion of patients (3, 4). Furthermore, clinical practice shows that after failure of the basic available modalities (anti-CD38 monoclonal antibodies, proteasome inhibitors, immunomodulatory agents, high-dose chemotherapy with autologous stem cell support, radiotherapy), the risk of aggressive forms of the disease, manifested mainly in the form of EMP, increases in connection with clonal development in the tumour population in the late stages (1). In the case described, this occurred after the first line of therapy, which included all above-mentioned therapeutic modalities. These were repeatedly applied in various combinations in the subsequent course of treatment, always with good but only short-term effect. Therefore, the first bispecific anti-BCMA antibody, teclistamab, which showed promising long-term effects, was chosen as the sixth line of treatment. Unfortunately, this had to be ended due to a prolonged COVID-19 infection, and the subsequent relapse took the form of EMP for the second time. The resumed treatment with the original bispecific antibody had no effect and was therefore discontinued after one month. The subsequent seventh line of treatment with a second bispecific antibody, whose effect in refractory disease has been repeatedly demonstrated (13), had only a limited effect in this case, as mentioned above. This situation led to a multidisciplinary consideration and the decision to proceed with this highly non-standard solution, albeit with little hope of achieving a curative effect.

This premise was confirmed over time. The total load of malignant cells in such advanced form of EMP was so massive that both conventional therapeutic procedures and cytoreductive surgery performed to this extent were unable to achieve an effective curative effect. From this point of view, this attempt at surgical cytoreduction must be considered unsuccessful.

From a technical point of view, however, we can state that the goal set from a surgical perspective was achieved. The nature and extent of the surgical procedure were carefully considered to achieve the maximum possible reduction of tumour masses with limiting periprocedural risks according to the patient's overall condition. To achieve this goal, numerous comprehensive measures were adopted and successfully applied. As a result, the surgical procedure itself and the postoperative period proceeded according to plan and without major complications, resulting in complete and successful healing of the surgical wound. The therapeutic attempt did not represent an unreasonable burden for the patient, did not worsen his quality of life, nor did it worsen his long-term prognosis.

If we are to ask ourselves whether CRS is a potentially promising method in the therapeutic algorithm for EMP in the future, it is obviously impossible to answer this question based solely on this case report. If we limit our perspective strictly to the described case, the question arises as to whether a better therapeutic effect would be achieved if the remaining EMP lesions had more favourable anatomical conditions, which would allow more extensive yet safe cytoreductive procedure in a single session. However, in the absence of substantiated data, this consideration would also be purely speculative. On the other hand, some published papers suggest that surgical treatment combined with conventional modalities, like chemotherapy, biological therapy or radiotherapy, could be quite effective in the solitary and operable lesions of EMP with none or less advanced systemic involvement (6, 9–11).

## Conclusion

Although the multiple myeloma and more specifically extramedullary plasmacytoma is not a common unit in the portfolio of surgeon's expertise, for the wide surgical public it is definitely important to be educated about the general principles of the treatment for the cases in which the surgical procedure is considered as a therapeutic option. In relation to this statement, it can be said about this kind of surgical procedure, that, assuming careful consideration of indications within the framework of interdisciplinary cooperation, taking into account the stage and extent of the disease, the location of the lesions, response to systemic therapy and the patient's overall condition, this may be an available, undemanding, safe procedure that is reproducible and does not exceed the capabilities of a standard surgical facility.

## Patient perspective

Patient gave his consent for surgical procedure as a last therapeutic attempt in the treatment of his underlying disease. He was fully informed about the nature of the procedure, risks, potential benefits and his long-term prognosis. In the time, when it was obvious that all therapeutic attempts were exhausted, patient himself asked for complete discontinuation of treatment, because he wished to spend the rest of his time calmly and peacefully. The patient died on 27 September 2025.

## Data Availability

The original contributions presented in the study are included in the article/Supplementary Material, further inquiries can be directed to the corresponding author.
